# Toxin-neutralizing antibodies protect against *Clostridium perfringens*-induced necrosis in an intestinal loop model for bovine necrohemorrhagic enteritis

**DOI:** 10.1186/s12917-016-0730-8

**Published:** 2016-06-13

**Authors:** Evy Goossens, Stefanie Verherstraeten, Bonnie R. Valgaeren, Bart Pardon, Leen Timbermont, Stijn Schauvliege, Diego Rodrigo-Mocholí, Freddy Haesebrouck, Richard Ducatelle, Piet R. Deprez, Filip Van Immerseel

**Affiliations:** Department of Pathology, Bacteriology and Avian Diseases, Faculty of Veterinary Medicine, Ghent University, Salisburylaan 133, B-9820 Merelbeke, Belgium; Department of Internal Medicine and Clinical Biology of Large Animals, Faculty of Veterinary Medicine, Ghent University, Salisburylaan 133, B-9820 Merelbeke, Belgium; Department of Surgery and Anesthesia of Domestic Animals, Faculty of Veterinary Medicine, Ghent University, Salisburylaan 133, B-9820 Merelbeke, Belgium

**Keywords:** Bovine necrohemorrhagic enteritis, *Clostridium perfringens*, Neutralizing antibodies, Alpha toxin, Perfringolysin O

## Abstract

**Background:**

Bovine necrohemorrhagic enteritis is caused by *Clostridium perfringens* type A. Due to the rapid progress and fatal outcome of the disease, vaccination would be of high value. In this study, *C. perfringens* toxins, either as native toxins or after formaldehyde inactivation, were evaluated as possible vaccine antigens. We determined whether antisera raised in calves against these toxins were able to protect against *C. perfringens* challenge in an intestinal loop model for bovine necrohemorrhagic enteritis.

**Results:**

Alpha toxin and perfringolysin O were identified as the most immunogenic proteins in the vaccine preparations. All vaccines evoked a high antibody response against the causative toxins, alpha toxin and perfringolysin O, as detected by ELISA. All antibodies were able to inhibit the activity of alpha toxin and perfringolysin O in vitro. However, the antibodies raised against the native toxins were more inhibitory to the *C. perfringens-*induced cytotoxicity (as tested on bovine endothelial cells) and only these antibodies protected against *C. perfringens* challenge in the intestinal loop model.

**Conclusion:**

Although immunization of calves with both native and formaldehyde inactivated toxins resulted in high antibody titers against alpha toxin and perfringolysin O, only antibodies raised against native toxins protect against *C. perfringens* challenge in an intestinal loop model for bovine necrohemorrhagic enteritis.

## Background

The ubiquitous, spore forming, Gram-positive bacterium *Clostridium perfringens* is considered to be the most widespread pathogenic bacterium in the world [1–4]. It can cause a wide range of diseases including, amongst others, gas gangrene in man and necrohemorrhagic enteritis in suckling and veal calves [5–8]. Most of these diseases follow a very rapid, often fatal course. Therefore, curative treatment is difficult and control must rely on preventive measures, including vaccination. Virulence properties of different *C. perfringens* strains are largely determined by their ability to secrete a variety of proteinaceous toxins and enzymes, which can cause different forms of tissue damage [2–4, 9]. Alpha toxin and perfringolysin O have been identified as the principal toxins involved in the pathogenesis of both *C. perfringens-*induced gas gangrene and bovine necrohemorrhagic enteritis [10, 11]. These toxins exert different effects in both diseases. Bovine necrohemorrhagic enteritis is characterized by congestion of the capillaries, hemorrhages and inflammation. This is in contrast to gas gangrene, where these toxins lead to tissue necrosis, thrombosis and lack of leukocyte infiltration at the site of infection [10–12]. It is well known that humoral antibodies against secreted proteinaceous virulence factors of *C. perfringens* can be protective, as shown in different animal models. As the enzymes and toxins of *C. perfringens* are highly destructive to tissues, vaccines against a variety of clostridial diseases have been developed using the denatured proteins [13–15]. Despite the usefulness of formaldehyde toxoids for other *C. perfringens-*associated diseases, there is controversy about the efficacy of such vaccines for gas gangrene, as opposed to crude toxin preparations [8, 16–18]. In addition, multivalent clostridial vaccines based on formaldehyde inactivated exotoxins derived from culture supernatant are commercially available for domestic livestock, including bovines, but no studies on their efficacy for necrohemorrhagic enteritis in calves are available.

The objective of the present study was to evaluate whether antibodies against *C. perfringens* toxins could protect against the development of necrotic lesions in the intestine. Therefore, calves were immunized with native *C. perfringens* toxins. To evaluate whether we could eliminate the undesired toxin activity, but conserve the immune-protective potential, a previously described, modified formaldehyde treatment was also tested [19]. Also a commercial formaldehyde inactivated multivalent clostridial vaccine was used. As necrohemorrhagic enteritis in veal calves is an unpredictable event and experimental reproduction of the disease is difficult, the neutralizing activity of the antibodies was evaluated in a previously developed intestinal loop model [20]. To further unravel the mechanism of protection, the inhibitory effect of the evoked antibodies on *C. perfringens-*induced cytotoxicity to bovine endothelial cells was evaluated and the toxin-neutralizing capacity against alpha toxin and perfringolysin O was analyzed.

## Results

### Western blot analysis

**Fig. 1:**
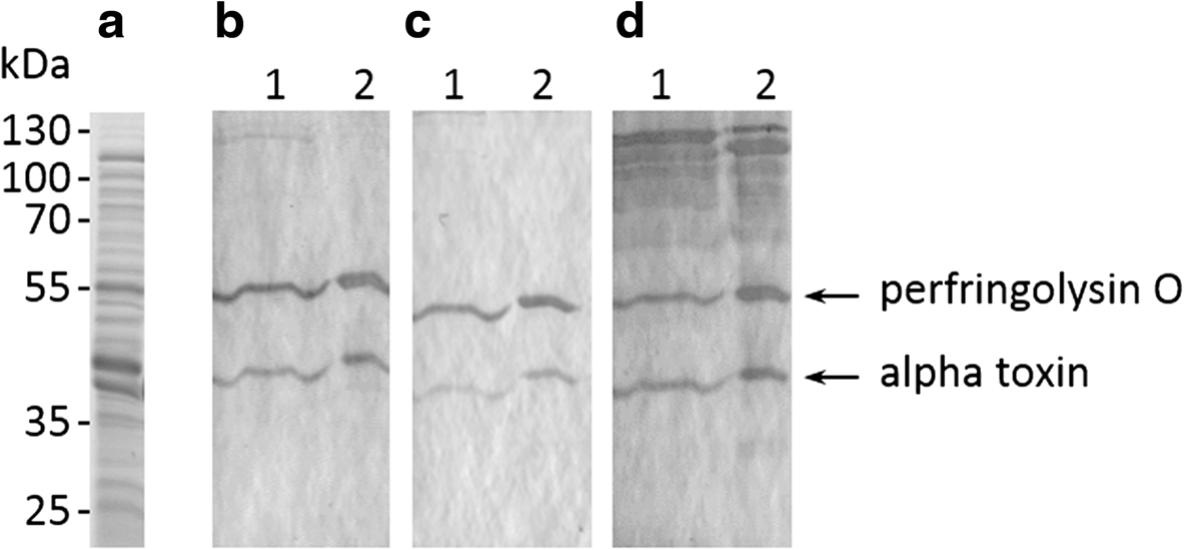
Western blot analysis of the immune sera. **a** SDS-PAGE of the *C. perfringens* toxin preparation after Coomassie staining. **b**-**d** Representative Western blots showing the immunoreactivity towards crude *C. perfringens* supernatants (lane 1) and the *C. perfringens* toxin preparation (lane 2). The immune sera from calves vaccinated with native toxins (**b**) and formaldehyde inactivated, L-lysine protected *C. perfringens* toxins (**c**) detect only two proteins, whereas the commercial formaldehyde inactivated multivalent clostridial vaccine (**d**) reacts with multiple proteins. The blots shown are representative pictures of one out of three experiments

The proteins in the *C. perfringens* toxin preparation were visualized by SDS-PAGE (Fig. 1a). In the vaccinated calves, the production of circulating antibodies against *C. perfringens* supernatant and the *C. perfringens* toxin preparation was analyzed by western blot in three separate experiments (Fig. 1). No immune reaction was detected in the sera before immunization (data not shown). Sera obtained from calves six weeks after initial vaccination with either native toxins or the L-lysine protected, formaldehyde inactivated toxins, revealed immunoreactivity towards two proteins. Immune sera from calves vaccinated with the commercial formaldehyde inactivated clostridial vaccine showed immunoreactivity towards more proteins. The two proteins that were immunoreactive with antisera raised against all vaccine preparations were further identified as alpha toxin and perfringolysin O by MALDI analysis.

### ELISA

In the vaccinated calves, the production of circulating antibodies directed against alpha toxin and perfringolysin O was also monitored by ELISA. No antibodies against alpha toxin or perfringolysin O were detected in the sera before immunization. In all calves a strong antibody response against both alpha toxin and perfringolysin O was detected 6 weeks after initial immunization. Calves vaccinated with the native *C. perfringens* toxins showed the highest antibody titers, whereas vaccination with formaldehyde inactivated toxins (either L-lysine protected or commercial inactivation) resulted in a more variable immune response (Table 1).

**Table 1:** Calves were immunized with either a *C. perfringens* toxin preparation (native toxins), L-lysine protected, formaldehyde inactivated *C. perfringens* toxins (L-lysine/formaldehyde toxoid) or a commercial multivalent formaldehyde inactivated clostridial vaccine

Vaccine	Anti-alpha toxin titer	Anti-perfringolysin O titer
Native toxins	64.44 ± 0.22	25600 ± 0
L-lysine/formaldehyde toxoid	24.26 ± 2.96	16000 ± 9600
Commercial formaldehyde vaccine	45.14 ± 20.42	4800 ± 1600

The anti-alpha toxin and perfringolysin O response was measured by ELISA. The data represent antibody titers (mean ± standard error of the means), six weeks after initial immunization

### Protective effect of antisera against *C. perfringens*-induced necrosis in an intestinal loop model

**Fig. 2:**
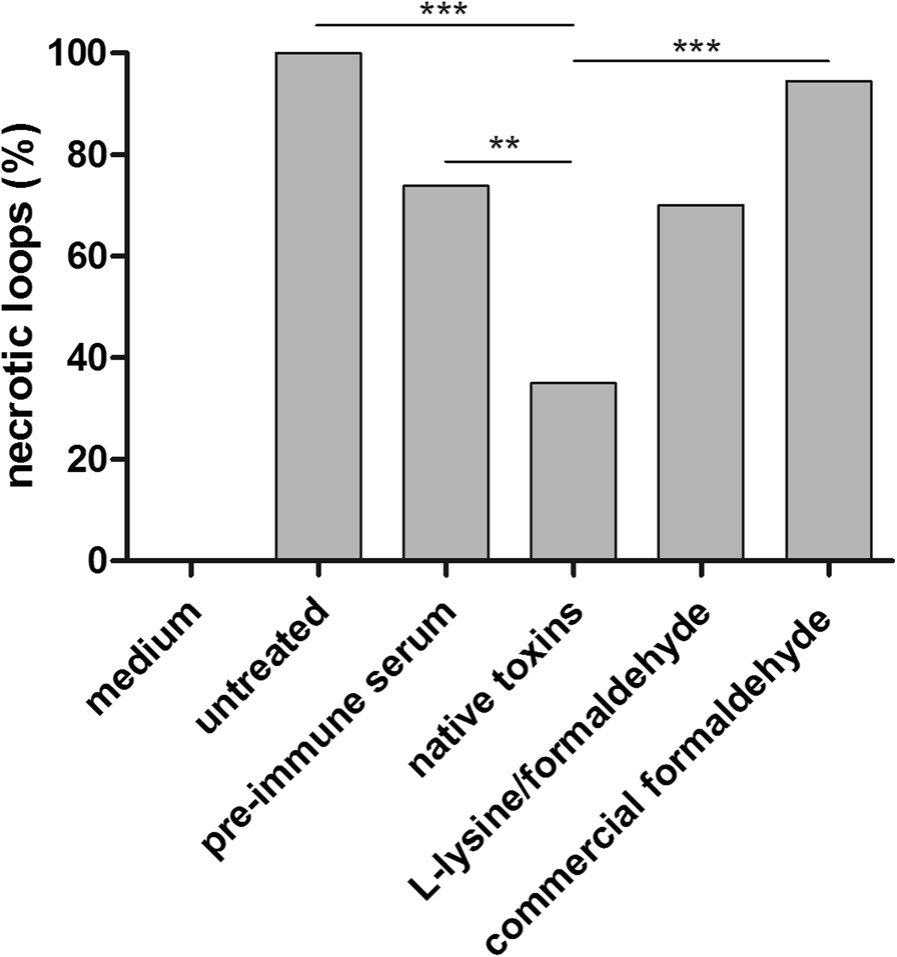
Neutralization of the lesion-inducing potential of *C. perfringens*. The graph represents the percentage of ligated intestinal loops in which necrotic lesions were present after 5 h of incubation with sterile culture medium (*n* = 20), *C. perfringens* alone (untreated, *n* = 20) or *C. perfringens* in combination with naive sera (pre-immune serum, *n* = 60), antiserum to *C. perfringens* toxins (native toxins, *n* = 20), formaldehyde inactivated, L-lysine protected *C. perfringens* toxins (L-lysine/formaldehyde, *n* = 20) and commercial formaldehyde inactivated multivalent clostridial vaccine (commercial formaldehyde, *n* = 20). ** 0.001 ≤ *p* < 0.01 or *** *p* < 0.001

**Fig. 3:**
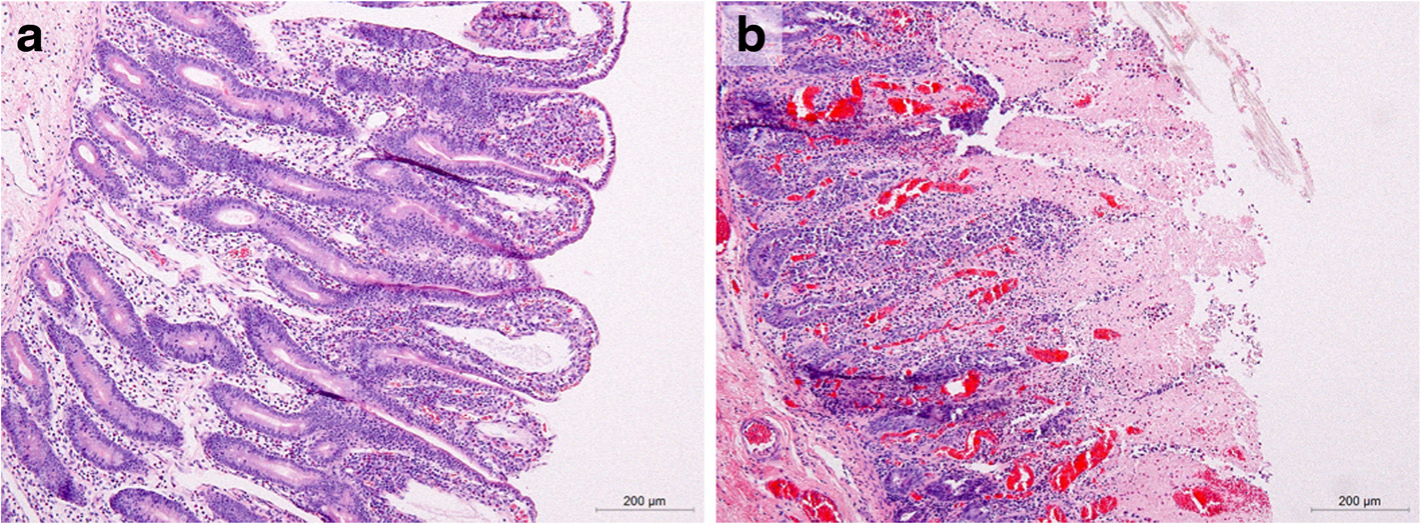
*C. perfringens*-induced necrosis in experimentally infected intestinal loops in calves. **a** Representative histological section from an intestinal loop without necrotic lesions. This loop was injected with *C. perfringens* in combination with antiserum to native *C. perfringens* toxins. **b** Representative section from an intestinal loop from the same calf, showing hemorrhage and extensive necrosis of the villi. This loops was injected with *C. perfringens* in combination with naïve immune serum

The potential of the antisera, derived after vaccination of calves with the respective vaccines, to inhibit *C. perfringens*-induced necrosis, was evaluated in an intestinal loop assay. All positive control loops inoculated with *C. perfringens* developed necrosis. Injection of loops with *C. perfringens* together with sera from naive calves (pre-immune sera) also resulted in a high percentage of necrotic loops. Injection of *C. perfringens* together with antisera raised against native toxins resulted in significantly fewer necrotic loops as compared to the positive control loops (*p* < 0.001) and the loops injected with the pre-immune sera (*p* < 0.01). Antisera raised against formaldehyde inactivated toxoid (either L-lysine protected or commercial) were unable to significantly neutralize the necrosis-inducing activity of *C. perfringens* (Figs. 2 and 3).

### Neutralization of alpha toxin and perfringolysin O activity in vitro

The inhibitory capacity of the sera towards alpha toxin and perfringolysin O activities was further examined using recombinant toxins. All antisera decreased the activity of alpha toxin in vitro (Table 2). Up to a final dilution of 409.8 the antisera against the native toxins neutralized 50 % of the alpha toxin activity. To the contrary, in order to obtain the same inhibition of alpha toxin activity, antisera against L-lysine protected, formaldehyde inactivated toxoid or against the commercial formaldehyde inactivated clostridial vaccine could only be diluted up to final dilutions of 80.47 or 22.39, respectively.

**Table 2:** In vitro neutralization of biological activities of alpha toxin and perfringolysin O. Calves were immunized with either a *C. perfringens* toxin preparation (native toxins), L-lysine protected, formaldehyde inactivated *C. perfringens* toxins (L-lysine/formaldehyde toxoid) or a commercial multivalent formaldehyde inactivated clostridial vaccine

	Inhibitory capacity (Mean ± SEM)	
Antiserum	Alpha toxin activity^a^	PFO activity^b^
Native toxins	409.8 ± 5.75	48.0 ± 0.0
L-lysine/formaldehyde toxoid	80.47 ± 46.93	72.0 ± 24.0
Commercial formaldehyde vaccine	22.39 ± 2.17	18.0 ± 6.0

^a^Neutralization of 10 μg/ml alpha toxin. The inhibitory capacity of the antiserum is expressed as the dilution that gives 50 % inhibition of the alpha toxin activity

^b^Neutralization of 2 μg/ml perfringolysin O. The inhibitory capacity of the antiserum is expressed as the highest dilution that inhibited perfringolysin O-induced hemolysis

Alpha toxin activity was determined by measuring its lecithinase activity on egg yolk lipoproteins. Perfringolysin O (PFO) activity was determined by measuring the hemolysis of horse erythrocytes

The hemolytic activity of perfringolysin O towards equine erythrocytes in vitro was decreased by all antisera (Table 2). Up to a final dilution of 18 either antiserum inhibited the perfringolysin O activity completely. This neutralizing ability of the sera was observed up to a final dilution of 48 for the anti-native toxins antisera, a final dilution of 72 for the anti-L-lysine protected, formaldehyde inactivated toxoid or a final dilution of 18 when the antiserum obtained after vaccination with the commercial vaccine was used. The pre-immune sera had no effect on the alpha toxin or perfringolysin O activity in vitro.

### Neutralization of *C. perfringens* cytotoxicity to bovine endothelial cells

To determine whether the cytotoxic activity of *C. perfringens* could be inhibited by the antisera to the vaccines, *C. perfringens* supernatants were incubated with serial dilutions of the antisera. Exposure of the endothelial cells to untreated *C. perfringens* supernatant resulted in 100 % cell death. More than 80 % cell viability could be measured by pre-incubation of the *C. perfringens* supernatant with a 32-fold dilution of the native toxins antiserum. At this concentration, neither the antisera raised against L-lysine protected, formaldehyde inactivated toxins nor the antisera raised against the commercial formaldehyde inactivated clostridial vaccine had an effect on the cytotoxicity (Table 3). The pre-immune sera had no effect on the *C. perfringens* cytotoxicity.

**Table 3:** In vitro neutralization of *C. perfringens* cytotoxicity. Calves were immunized with either a *C. perfringens* toxin preparation (native toxins), L-lysine protected, formaldehyde inactivated *C. perfringens* toxins (L-lysine/formaldehyde toxoid) or a commercial multivalent formaldehyde inactivated clostridial vaccine

Antiserum	Inhibitory capacity (Mean ± SEM)
Native toxins	32.00 ± 0.0
L-lysine/formaldehyde toxoid	9.00 ± 7.0
Commercial formaldehyde vaccine	4.00 ± 0.0

The cytotoxicity of *C. perfringens* supernatant to primary bovine endothelial cells was measured using a neutral red uptake (NRU) assay. The inhibitory capacity of the antiserum is expressed as the highest dilution that yields 80 % cell viability

## Discussion

Necrohemorrhagic enteritis caused by *C. perfringens* in suckling and veal calves is characterized by sudden death. Due to the very rapid course of the disease, curative treatment is not possible and therefore, protection by vaccination would be of high value. The virulence of *C. perfringens* is due to the many extracellular toxins it produces*.* In this study we showed that toxin neutralizing antibodies protect against *C. perfringens-*induced necrotic lesions in an intestinal loop assay and are able to prevent endothelial damage. Western blot analysis revealed antibodies towards alpha toxin and perfringolysin O as the most abundant antibodies in the immune sera from calves vaccinated against *C. perfringens* toxins.

We previously reported congestion and leakage of the capillaries as an early event in the pathogenesis of necrohemorrhagic enteritis as shown in an intestinal loop assay [20]. Furthermore we showed that alpha toxin and perfringolysin O may exert their effect by directly targeting the endothelial cells [10]. This points towards endothelial damage as a key event in the pathogenesis of bovine necrohemorrhagic enteritis. Indeed, in the present study antisera which protected against *C. perfringens-*induced cytotoxicity to bovine endothelial cells also offered protection against *C. perfringens-*associated necrosis in an intestinal loop assay. Moreover, the protective antisera were shown to inhibit the activity of alpha toxin and perfringolysin O, which further underscores the roles of these toxins in the pathogenesis of bovine necrohemorrhagic enteritis. It can, however, not be ruled out that antibodies induced against other substances present in the vaccines also played a role in the protection observed in the intestinal loop model.

Formaldehyde inactivation of *C. perfringens* toxins diminished their capacity to induce protective antibodies. Antisera raised against L-lysine protected, formaldehyde inactivated *C. perfringens* toxins were also not protective in the intestinal loop model. This result is in disagreement with previous studies showing high antigenicity, low toxicity, and protection in mice that were immunized with L-lysine protected, formaldehyde inactivated toxoid and subsequently challenged with lethal doses of *C. perfringens* [19, 21]*.* In the present study we demonstrated that vaccinations with *C. perfringens* toxins, either in their native forms or as formaldehyde inactivated toxoids, all resulted in high antibody responses as detected by ELISA. However, only serum derived from animals immunized with the native toxins offered protection against necrosis in an intestinal loop assay. There is thus a discrepancy between the antibody titers against formaldehyde inactivated *C. perfringens* toxins measured by ELISA and the protective capacity of these antibodies in the intestinal loop model. Nevertheless, the value of vaccines based on formaldehyde inactivated *C. perfringens* toxins has been demonstrated for diseases associated with toxins other than alpha toxin and perfringolysin O [22–25]. This suggests that the protective immunogenicity of other *C. perfringens* toxins, such as, amongst others, NetB and epsilon toxin, is not affected by formaldehyde inactivation.

Although the use of *C. perfringens* native toxins represents an efficient strategy for vaccine development, active toxins cannot be regarded as safe. Therefore methods for the development of toxoids other than formaldehyde inactivation are needed. Possible strategies include the use of genetically modified toxoids based on site-directed mutants with reduced toxic activity or the use of immunologically active fragments of the essential toxins. Immunization with the carboxy-terminal domain of alpha toxin has previously been shown to provide protection in a mouse model against *C. perfringens* gas gangrene and may be a good candidate for development of a vaccine against bovine necrohemorrhagic enteritis [21, 26]. The identification of the structural elements responsible for membrane interaction of perfringolysin O provides opportunities for the development of non-toxic site-directed mutants as alternatives for native perfringolysin O [27].

In order to obtain the ultimate evidence that vaccination against *C. perfringens* toxins protects against bovine necrohemorrhagic enteritis, field trials need to be performed. However, since necrohemorrhagic enteritis is a low incidence disease, this would be a huge cost and more evidence concerning the immune-protective potential of the antisera is needed before considering this type of trial. Unfortunately, no in vivo model to validate the protective immune-potential of the candidate vaccines against bovine necrohemorrhagic enteritis is available. Niilo and colleagues were able to induce a mild diarrhea in cattle inoculated intraduodenally or per os with *C. perfringens* type A cultures, but no necrohemorrhagic enteritis was established [28]. Also we were unable to develop a reliable model of bovine necrohemorrhagic enteritis after per os or intraduodenal administration of *C. perfringens* type A cultures (unpublished results).

## Conclusion

This study showed that toxin-neutralizing antibodies protect against *C. perfringens* challenge in an intestinal loop model for bovine necrohemorrhagic enteritis. Immunization of calves with either native or formaldehyde inactivated toxins resulted in a strong immune response against alpha toxin and perfringolysin O, but only antibodies raised against native toxins were protective in the intestinal loop model. Therefore it seems that, at least for alpha toxin mediated diseases, antibody titers detected by ELISA are not a guarantee for protection, even if protection against the disease is antibody mediated.

## Methods

### Vaccine preparation and immunization

*C. perfringens* toxin preparation (P4039, Sigma-Aldrich, Bornem, Belgium) was either used as native toxin or treated with formaldehyde to generate a formaldehyde toxoid. Inactivation was obtained by adding a combination of 0.4 % formaldehyde solution (Sigma-Aldrich) and 0.05 M L-lysine (Sigma-Aldrich) and incubation at 37 °C for two days. The addition of 0.05 M L-lysine has previously been shown to preserve the antigenicity of alpha toxin during toxoid formation [19]. Inactivation of alpha toxin was confirmed by spotting 5 μl drops on 2 % egg yolk Columbia agar plates (Oxoid, Wesel, Germany), followed by incubation for 16 h at 37 °C [29]. Native and formaldehyde inactivated toxins were formulated with the adjuvant Quil A (Brenntag Biosector, Frederikssund, Denmark) at a final concentration of 350 μg antigen and 750 μg Quil A in 1.5 ml phosphate buffered saline (PBS) per animal and filter-sterilized using a 0.2 μm filter. A standard formalin inactivated multivalent commercial vaccine was used according to the manufacturer’s instructions (Covexin 10®, Zoetis, Louvain-la-Neuve, Belgium).

For immunization six 2-months old male Holstein Friesian calves were used. The calves were purchased from a local tradesman which collects dairy calves from herds in Eastern Flanders. They were housed on straw and received water and hay *at libitum*, and concentrates adjusted to the body weight.

For each antigen, two calves were immunized subcutaneously in the neck. The calves received a primer vaccination at the age of two months, with booster immunizations 14 and 28 days later. No strong adverse reactions were observed. Although no fever (>39.5 °C) was induced, all calves experienced a mild hyperthermia for two days following the vaccination. As described in the drug information leaflet of the commercial vaccine, localized swelling occurred at the site of injection. This effect was more pronounced in the calves vaccinated with the commercial formaldehyde inactivated clostridial vaccine (7–10 cm diameter) as compared to the calves vaccinated with either native toxins or the L-lysine protected, formaldehyde inactivated toxins (0–6 cm diameter). Blood samples were taken before primer vaccination and two weeks after the final booster vaccination.

### SDS-PAGE and Western Blot

The proteins present in the toxin preparation were visualized on a 12 % SDS-PAGE followed by Coomassie Briliant Blue staining (Sigma-Aldrich). For the Western Blot analysis, 16 μl of cell-free supernatants of the *C. perfringens* strain JIR325 (10x concentrated using Vivaspin, Sartorium Stedim Biotech GmbH, Goettingen, Germany) or 6 μg of the *C. perfringens* toxin preparation were loaded on a 12 % SDS-PAGE. The proteins from the gel were transferred to nitrocellulose membranes of 0.45 μm pore size. Non-specific binding to the blots was blocked with 5 % skimmed milk powder in PBS, followed by overnight incubation at 4 °C with a 1/500 dilution of the immune sera collected two weeks after the final booster immunization. For this incubation step, the sera of the 2 animals that were vaccinated with a given vaccine preparation were pooled. Blots were washed with 0.1 % tween 20 in PBS and incubated for 1 h at room temperature with horseradish peroxidase-labelled rabbit-anti-bovine IgG (Sigma-Aldrich). Blots were developed with CN/DAB substrate kit (Thermo Fisher Scientific, Rockford, USA). The test was performed in triplicate. The specific immunoreactive protein bands were identified in the parallel-run Coomassie stained gel followed by MALDI analysis.

### Enzyme-linked immunosorbent assay

The immune response following vaccination was also measured by ELISA using serum samples two weeks after the final booster immunization.

Alpha toxin-specific antibody levels were determined by the end-point dilution method using a blocking ELISA (*Clostridium perfringens* alpha toxin serological ELISA kit, Bio-X Diagnostics, Jemelle, Belgium). For each ELISA, sera were used at a dilution 1:50 and assays were performed in duplicate. The specific antibody level of the immune serum was expressed as the percent inhibition (% inhib) by means of the following formula: % inhib = [(OD neg – OD sample)/OD neg] * 100.

Perfringolysin O-specific antibody levels were measured using an indirect ELISA. Briefly, 96-well microtitre plates (Nunc MaxiSorp, Thermo Fisher Scientific) were coated with 20 μg recombinant perfringolysin O [30]. Non-specific binding was blocked with 1 % (w/v) bovine serum albumin (Sigma-Aldrich) in PBS. Two-fold dilutions of the sera ranging from a dilution of 1:50 to 1:51200 were added to the plates (100 μl of each dilution/well; in duplicate) and incubated for 2 h at 37 °C. Plates were washed with 0.1 % (v/v) Tween 20 in PBS and incubated for 1 h 30 min at 37 °C with horseradish peroxidase-labelled rabbit-anti-bovine IgG (Sigma-Aldrich). Bound conjugate was detected using the substrate 3,3′,5,5′-Tetramethylbenzidine (TMB) (Sigma-Aldrich). The reaction was blocked with H_2_SO_4_ and the absorbance was measured at 450 nm using a microplate reader (Multiscan MS, Thermo Labsystems, Helsinki, Finland). The end-point titer is expressed as the reciprocal of the last dilution that gave a reading of 0.1U above background (precolostral neonatal bovine serum).

### Intestinal loop model

To study the protection against *C. perfringens-*induced necrosis provided by the antisera from calves vaccinated with the vaccine preparations, four intestinal loop experiments were performed. Intestinal loop experiments were performed according to a previously described protocol using 4 healthy male Holstein Friesian calves, purchased from a local tradesman which collects dairy calves from herds in Eastern Flanders [20]. Briefly, the calves were anesthetized and the small intestine was exteriorized. Per calf 80 intestinal loops of approximately 10 cm were ligated in the jejunum and a 5 cm space was left between the loops. Only half of the loops were injected, thus each time leaving one intervening loop to avoid leakage between sampled loops. For each vaccine preparation individual pre- and post-vaccination sera of 2 calves were used in two intestinal loop experiments. Intestinal loops were inoculated with 20 ml of a wild-type strain (JIR325) in combination with 10 ml of 25 % commercial milk replacer suspended in sterile NaCl solution, resulting in a total volume of 30 ml which was the same across all treatments and control loops. Prior to inoculation pre- or post-immune serum derived from calves immunized with the different vaccine preparations was added to the NaCl solution containing milk replacer, to obtain a final concentration of 6 % serum (v/v). In each calf five intestinal loops per test serum were injected. Also an equal number of control loops without addition of serum were injected either with *C. perfringens* (positive control) or with sterile bacterial growth medium (negative control). After injection of the loops, the abdomen was closed and the calves were maintained under anesthesia. At 5-h post-inoculation intestinal biopsy samples were taken, after which the animals were euthanized. Samples were fixed in 4 % phosphate buffered formaldehyde. They were embedded in paraffin wax, sectioned and stained with hematoxylin-eosin. The sections were evaluated in a blinded manner by a board certified pathologist for presence of tissue necrosis (0 = absence of necrosis, 1 = necrotic lesions present).

### In vitro neutralization and cytotoxicity tests

#### Neutralization of alpha toxin activity on egg yolk lipoproteins in vitro

The alpha toxin activity was determined by its effect on egg yolk lipoproteins as previously described [31]. Therefore, fresh egg yolk was centrifuged (10,000 × g for 20 min at 4 °C) and diluted 1:10 in PBS. The ability of the sera to neutralize the alpha toxin activity was assessed by pre-incubating a two-fold dilution series of the sera (two wells per dilution) with a constant amount of alpha toxin (10 μg/ml recombinant alpha toxin in PBS solution) for 30 min at 37 °C prior to the addition of 10 % egg yolk emulsion. Recombinant alpha toxin was expressed in *E. coli* using the pBAD TOPO® TA Expression Kit (Invitrogen, Paisley, UK) followed by purification onto a Ni-sepharose column (His Gravitrap, GE Healthcare, Buckinghamshire, UK). After incubation of the plates at 37 °C for 1 h, the A_620_ was determined. Alpha toxin activity was indicated by the development of turbidity which results in an increase in absorbance. The inhibitory capacity of the antiserum was determined by applying a Hill function to the concentration-response data (GraphPad Prism 5, GraphPad Software, San Diego, CA, USA) and expressed as the dilution that inhibited 50 % of the alpha toxin activity. The test was performed in duplicate.

#### Neutralization of perfringolysin O activity in vitro

Perfringolysin O (PFO) activity was determined by measuring the hemolysis of horse erythrocytes using a doubling dilution assay as previously described [32]. The PFO titer is the reciprocal of the last dilution which showed complete hemolysis. Similar to the inhibition of the alpha toxin activities, the ability of sera to neutralize the PFO activity was assessed by pre-incubating a two-fold dilution series of the sera (two wells per dilution) with a constant amount of perfringolysin O (2 μg/ml recombinant perfringolysin O). Recombinant perfringolysin O was produced as previously described [33]. The inhibitory capacity of the antiserum was expressed as the highest dilution that inhibited perfringolysin O induced hemolysis. The test was performed in duplicate.

### Endothelial cell cytotoxicity assay

Primary bovine umbilical vein endothelial cells (BUVEC) were isolated from umbilical cord veins by an adaptation of the method of Jaffe et al. as performed previously [10, 34]. The toxicity of *C. perfringens* supernatant towards cultured bovine endothelial cells has been reported previously [10]. The ability of the antisera to neutralize the *C. perfringens* cytotoxicity towards BUVECs was determined using a Neutral Red Uptake assay (NRU) [35]. Therefore, a two-fold dilution series of the sera (100 % - 0.4 %) prepared in serum free cell culture medium was pre-incubated for 30 min at 37 °C with an equal amount of *C. perfringens* supernatant. Cells were treated with 100 μl of these supernatant-serum mixtures. The inhibitory capacity of the antiserum was expressed as the highest dilution that yielded 80 % cell viability. As a positive control, cells were treated with *C. perfringens* supernatant which was pre-incubated for 30 min with serum free cell culture medium. Untreated cells, incubated with serum free cell culture medium served as a negative control. The test was performed in duplicate.

### Statistical analysis

The 20 loops tested for each condition provided enough statistical power to detect a 40 % reduction in the development of necrotic lesions in the intestinal loop assay (95 % confidence, 80 % power) (Winepiscope 2.0).

The protective effect of the antisera in the intestinal loop assay as compared to the pre-immune sera and the untreated control loops were determined by a Fisher’s exact test (GraphPad Prism 5 software). Differences between groups were considered significant at *p* < 0.05.

## Abbreviations

BUVEC, bovine umbilical vein endothelial cells; ELISA, enzyme-linked immunosorbent assay; NRU assay, neutral red uptake assay; PBS, phosphate buffered saline; PFO, perfringolysin O; TMB, 3,3′,5,5′-Tetramethylbenzidine
